# New Drugs, Appliances, and Things Medical

**Published:** 1891-05-23

**Authors:** 


### NEW DRUGS, APPLIANCES, AND THINGS MEDICAL.

[All preparations, appliances, novelties, etc., of which a notice is desired, should be sent for The Editor, to care of The Manager, 140, Strand, London, W.C.]

### SCRUBB'S CLOUDY HOUSEHOLD AMMONIA.

This is a useful ammoniacal preparation of full strength, which the busy housewife will find always ready to hand. For cleansing purposes, whether for the wash-tub, or for plate, or for clothing, it will be found a decided acquisition to the store cupboard. A little added to the hot bath will aid in removing that excessive feeling of fatigue which prolonged exercise gives. We have long recommended ammonia in a foot bath of very hot water as the best restorative for the tired legs and feet of those who have to stand a greater portion of the day. The present preparation has yielded good results in our hands in this respect. We have no doubt it will prove equally useful in the treatment of gnat bites and insect stings of all sorts, and for the thousand and one purposes for which a liquid form of ammonia is required. The idea is ingenious, and we fancy that this is the only liquid ammonia in the field other than pharmaceutical preparations. It ought to prove a success. The offices of the firm who produce it are in Red Cross Street, E.C., and they will be pleased to forward samples to any medical man who will send a request for them to the office.

### ÆSCULAP WATER.

Among tried, proved, and safe remedies, Æsculap Water continues to hold its own. It is, indeed, if possible, still more approved and used by the medical profession than it has ever been. It well deserves this universal confidence. Æsculap has all the qualities which an aperient should possess. It is mild but effective, prompt in action, but not worrying; wholesome and safe. As a simple and pure saline it has no superior and few equals. The range of its use is very wide. Men, women, and children may alike take it, and under almost any circumstances. It is surprising that the springs do not become exhausted and that the water does not change in quality. We have made particular enquiries on this subject, and find that the results of analysis are practically constant. In the interests of those who need and value a mild and safe aperient we cannot but hope that Æsculap will continue to remain as effective, wholesome, and safe as it has always hitherto proved itself to be.

